# Supplementary material for: A bio-inspired coordination polymer as outstanding water oxidation catalyst via second coordination sphere engineering
Source: Nat Commun. 2019 Nov 7;10:5074. doi: 10.1038/s41467-019-13052-1 (PMC6838099; doi:10.1038/s41467-019-13052-1)
Supplement: Supplementary file 1 — Supplementary Information [file 41467_2019_13052_MOESM1_ESM.pdf]

Supplementary Information

A Bio-inspired Coordination Polymer as Outstanding Water Oxidation Catalyst via Second Coordination Sphere Engineering

Li et al.

Supplementary Figures

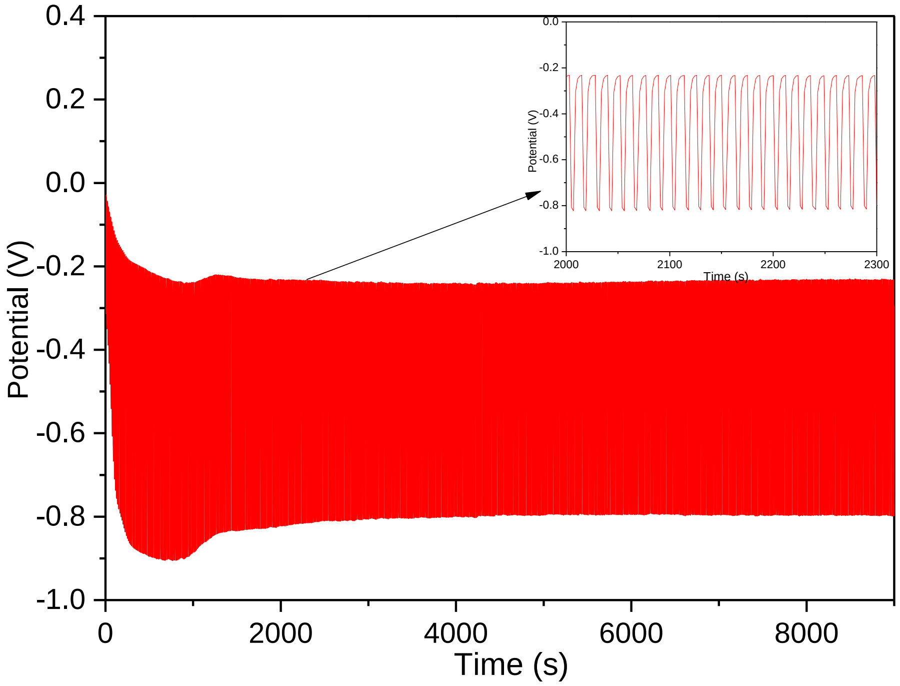

Supplementary Figure 1. The repeated double-current pulse chronopotentiometry (r-DCPC) process for **NiFeCP** films preparation.

The inset is part of an enlarged version, one r-DCPC cycle involved two current pulses at –3.0 mA cm^{-2} and 0 mA cm^{-2} for 5 s and 10 s, respectively.

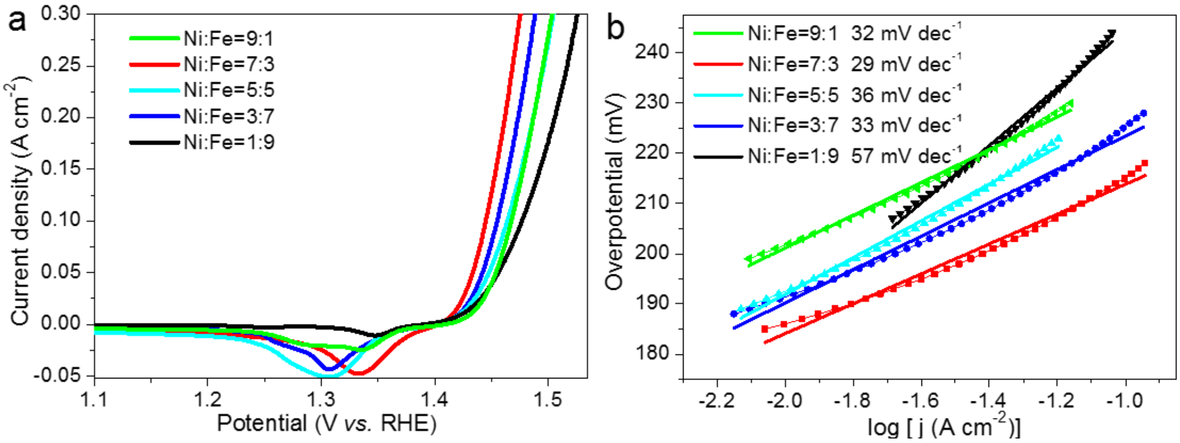

Supplementary Figure 2. Electrochemical characterizations for OER.

a Polarization curves of **NiFeCP/NF** with different proportions of Ni:Fe in the electrodeposition solution for OER, measured in 1.0 M KOH solution at a scan rate of 5 mV s^{-1}.

b Tafel plots for **NiFeCP/NF** with different proportions of Ni:Fe in the electrodeposition solution for OER, calculated from the data of **Supplementary Figure 2a**.

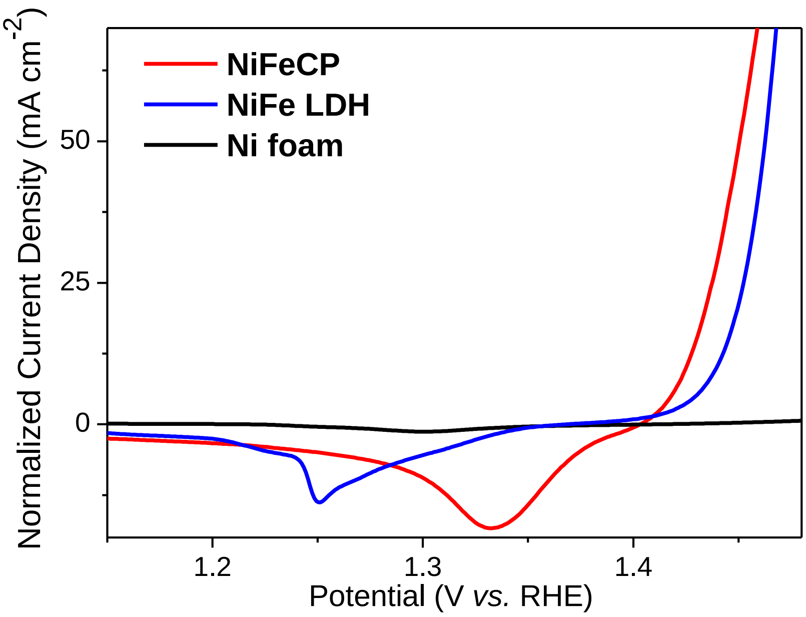

Supplementary Figure 3. Normalized LSV curves of **NiFeCP/NF** and **NiFe LDH/NF**.

The electrochemical active surface areas (ECSA) of **NiFeCP/NF** and **NiFe LDH/NF** electrodes were calculated as the following equation: ECSA = C_{dl}/4.23 mF cm^{-2}. The Ni foam with 1 cm^2 geometry surface area was used as reference with a double layer capacitance (C_{dl}) of 4.23 mF cm^{-2}. The electrochemical data (**Fig. 1a** in the main text) of **NiFeCP/NF** and **NiFe LDH/NF** are normalized to ECSA, respectively.

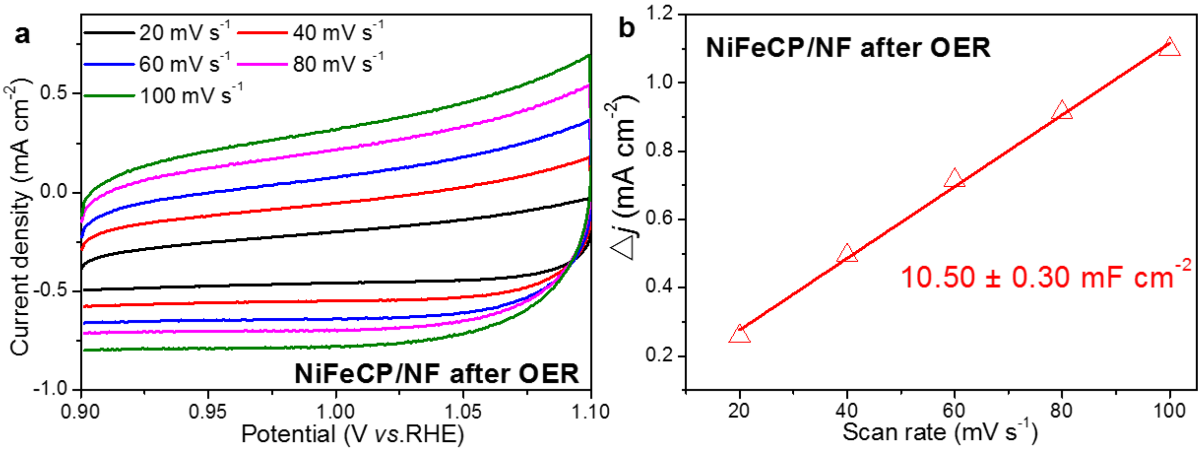

Supplementary Figure 4. Determination of ECSA for **NiFeCP/NF** after OER.

a Cyclic voltammetry (CV) curves of **NiFeCP/NF** electrode after 17 h of OER in 1 M KOH with different scan rates, the scanning potential range is from 0.9 V to 1.1 V vs RHE.

b Capacitance Δj ($|j_{charge} - j_{discharge}|$) versus the scan rates.

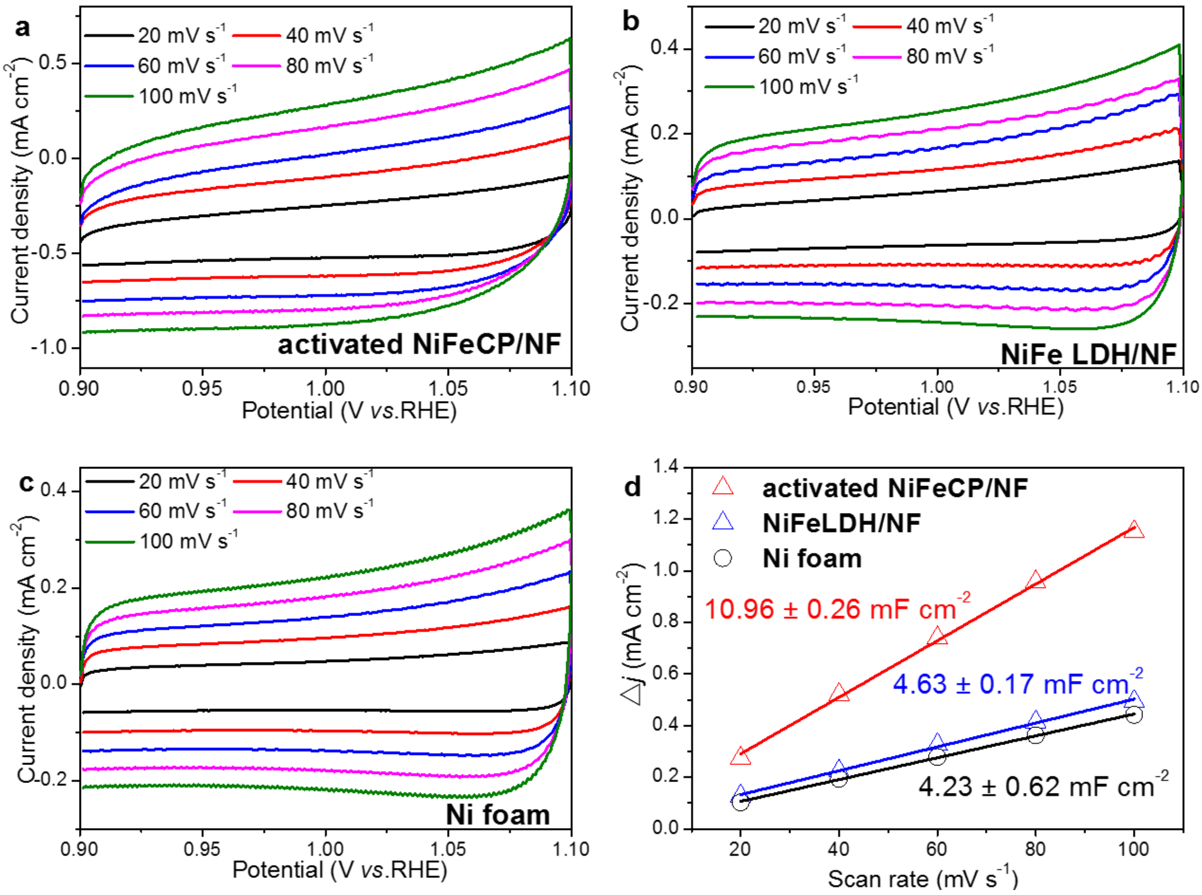

Supplementary Figure 5. Determination of the ECSA for the activated **NiFeCP/NF**, **NiFe LDH** and Ni foam.

a CV curves of activated **NiFeCP/NF** electrode in 1 M KOH with different scan rates at selected potential range.

b CV curves of **NiFe LDH/NF** electrode in 1 M KOH with different scan rates at selected potential range.

c CV curves of Ni foam electrode in 1 M KOH with different scan rates at selected potential range.

d The corresponding capacitance Δj ($|j_{charge}-j_{discharge}|$) versus the scan rates. The scanning potential range is from 0.9 V to 1.1 V vs RHE. ECSA of electrode was obtained from CV curves, in details, by plotting the Δj ($|j_{charge}-j_{discharge}|$) at Faradaic silence potential range against the scan rates, the linear slope is obtained, which is a positive correlation with the double-layer capacitance (C_{dl}), and been used to represent the corresponding ECSA.

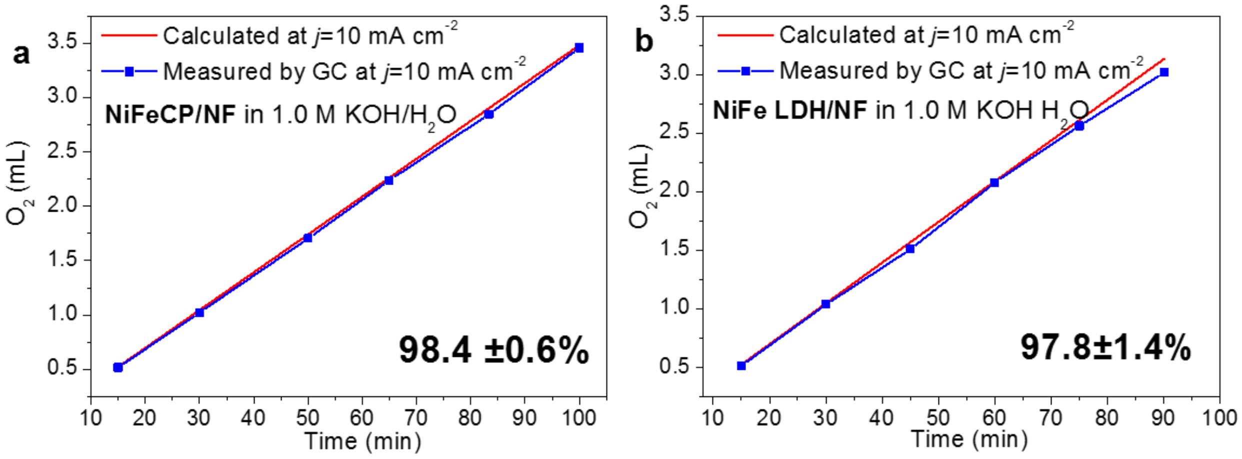

Supplementary Figure 6. The Faradaic efficiency of **a NiFeCP/NF** and **b NiFe LDH/NF** for OER in 1.0 M KOH H_2O.

Faradaic efficiency was determined by comparing the measured amount of oxygen gas and the theoretical value calculated on the basis of the transferred charge for **NiFeCP/NF** and **NiFe LDH/NF** at a current density of 10 mA cm^{-2}. The quantitative yields of **NiFeCP/NF** and **NiFe LDH/NF** were 98.4±0.6% and 97.8±1.4%, respectively.

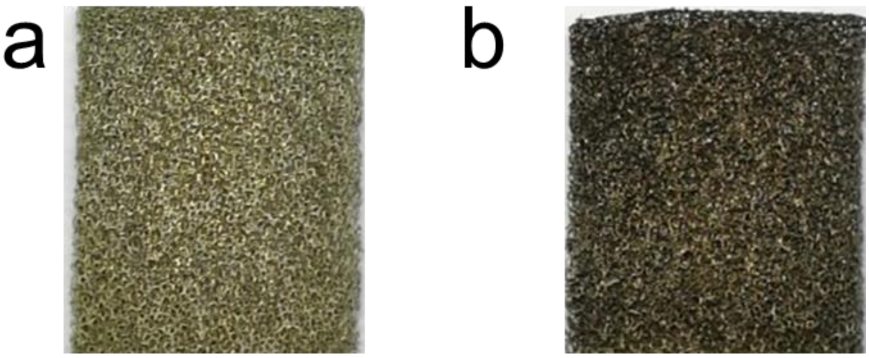

Supplementary Figure 7. The photograph of **NiFeCP/NF.**

a NiFeCP/NF as prepared.

b NiFeCP/NF after OER test.

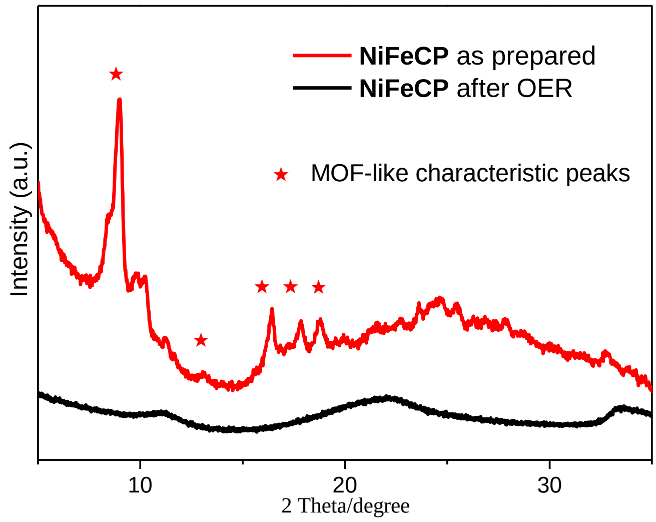

Supplementary Figure 8. XRD patterns of particles detached by sonication from as prepared **NiFeCP/NF** electrode and the electrode after OER test.

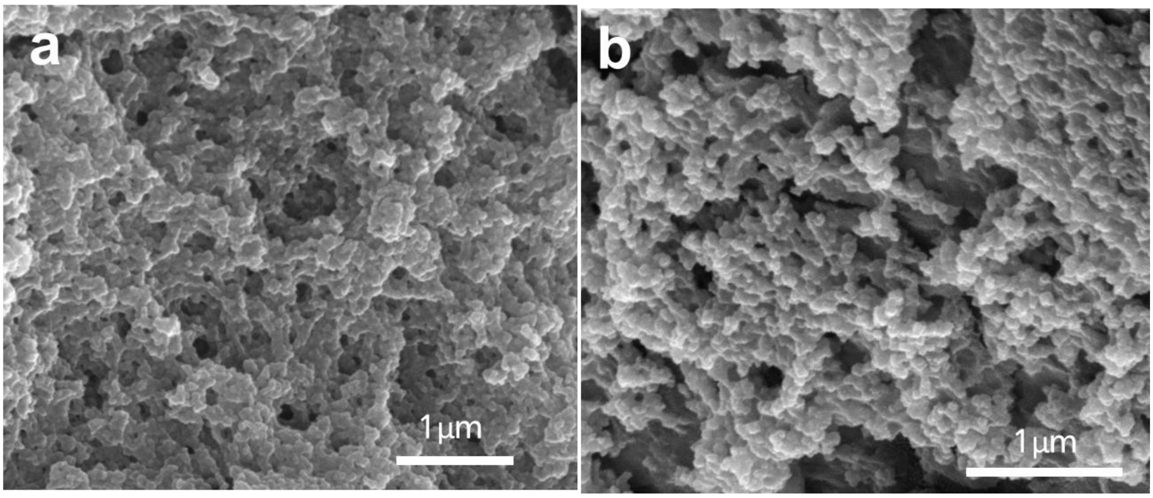

Supplementary Figure 9. SEM images of **NiFeCP/NF**.

a NiFeCP/NF as prepared.

b NiFeCP/NF after OER test.

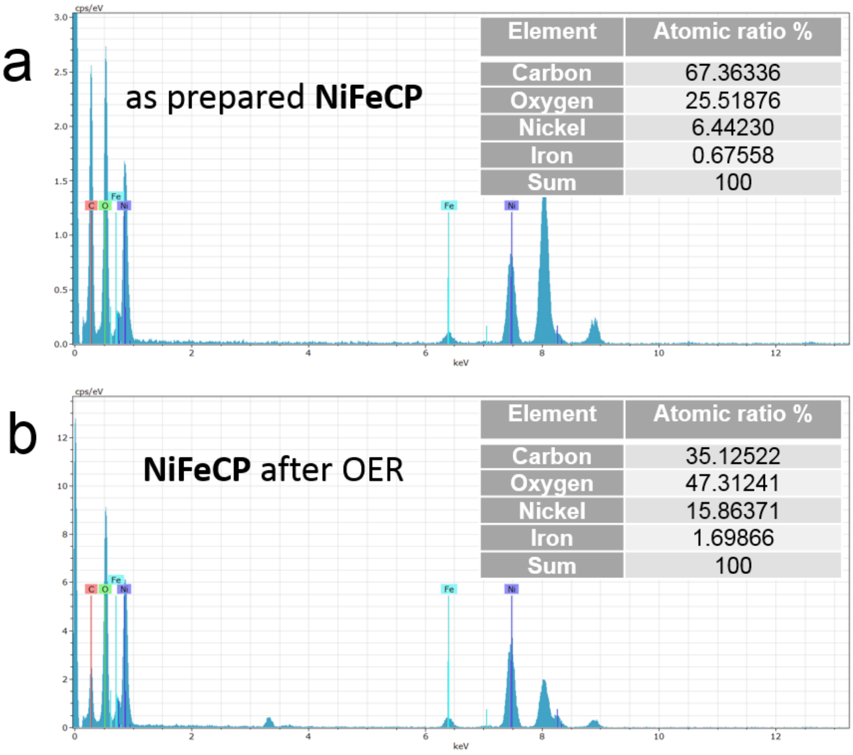

Supplementary Figure 10. The TEM-EDS spectra of **NiFeCP/NF**.

a Particles detached by sonication from as prepared **NiFeCP/NF** electrode.

b Particles detached by sonication from **NiFeCP/NF** after OER test.

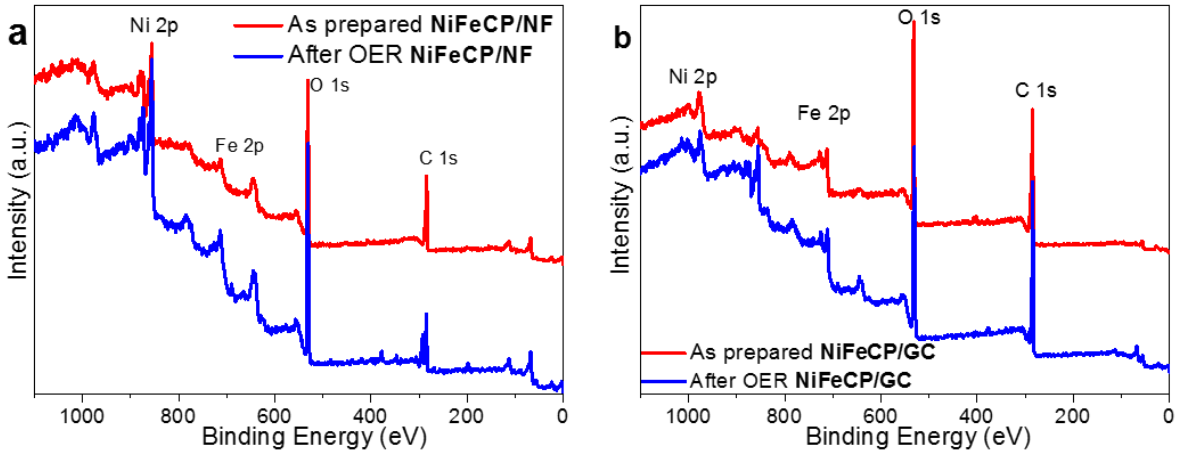

Supplementary Figure 11. XPS survey spectra of **NiFeCP** at different substrates.

a Survey spectrum XPS survey spectra of particles detached by sonication from as prepared **NiFeCP/NF** electrode and the electrode after OER test.

b Survey spectrum XPS survey spectra of particles detached by sonication from as prepared **NiFeCP/GC** electrode and the electrode after OER test.

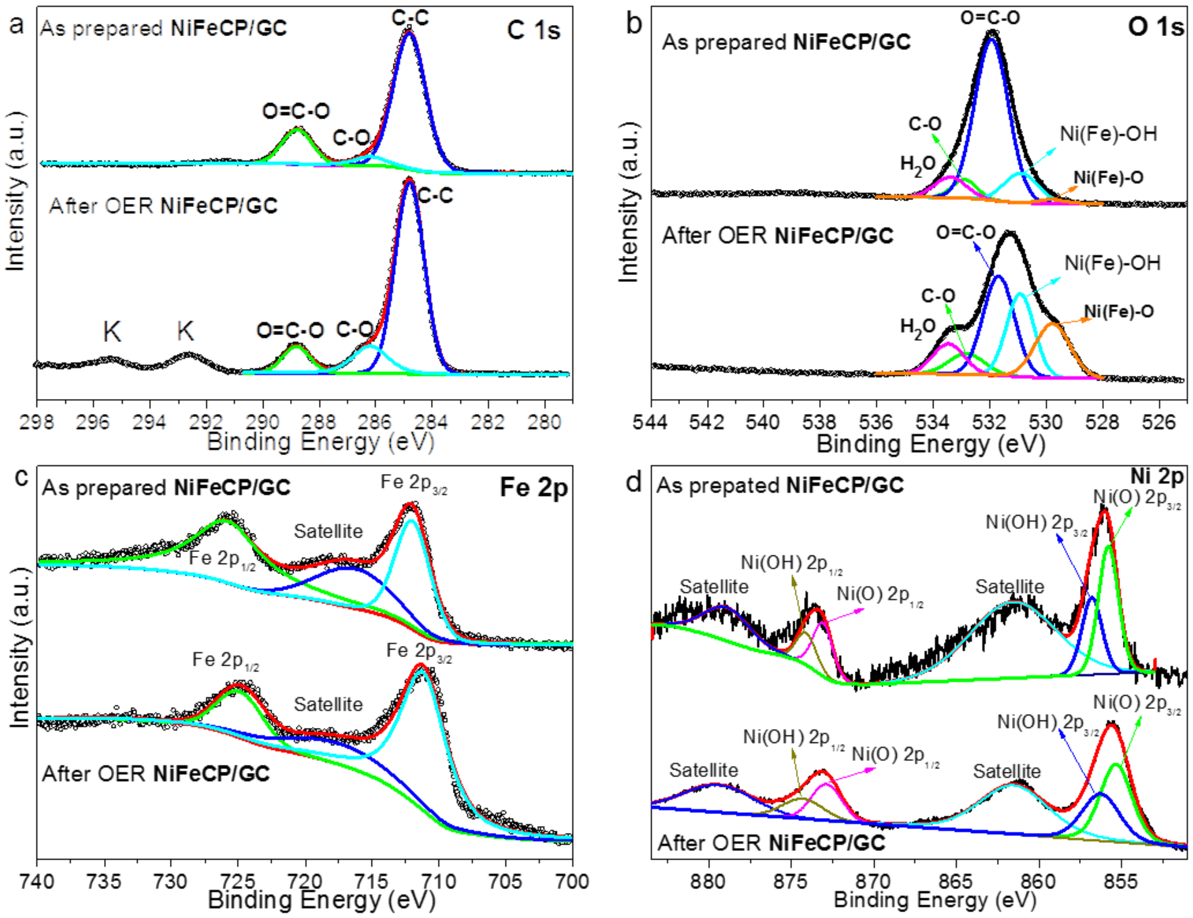

Supplementary Figure 12. High-resolution XPS spectra of **NiFeCP/GC.**

High-resolution XPS spectra of **a** C 1s, **b** O 1s, **c** Fe 2p, and **d** Ni 2p for particles detached by sonication from as prepared **NiFeCP/GC** and **NiFeCP/GC** after 5 h OER test. The ratio of the integrated area associated with the Ni-OH/NiO peaks increased from 6:10 to 7.2:10 after electrolysis for the **NiFeCP/GC** sample.

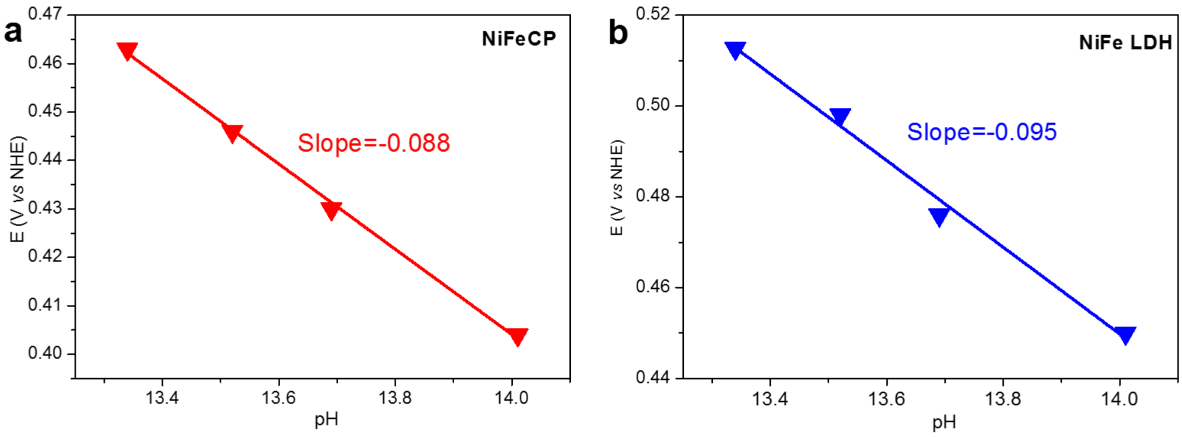

Supplementary Figure 13. Corresponding position (vs normal hydrogen electrode, NHE) of redox peaks for **a NiFeCP/NF** and **b NiFeLDH/NF.**

Data was extracted from LSV curves of **Fig. 5a** and **5b** in the main text.

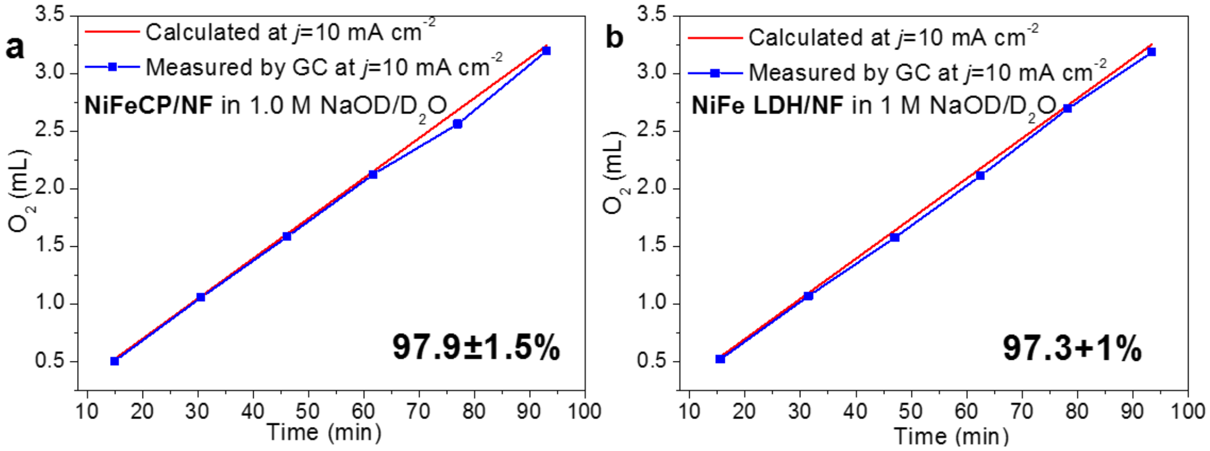

Supplementary Figure 14. The Faradaic efficiency of **a NiFeCP/NF** and **b NiFe LDH/NF** for OER in a 1.0 M NaOD D_2O solution.

Faradaic efficiency was determined by comparing the measured amount of oxygen gas and the theoretical value calculated on the basis of the transferred charge for **NiFeCP/NF** and **NiFe LDH/NF** at a current density of 10 mA cm^{-2}.

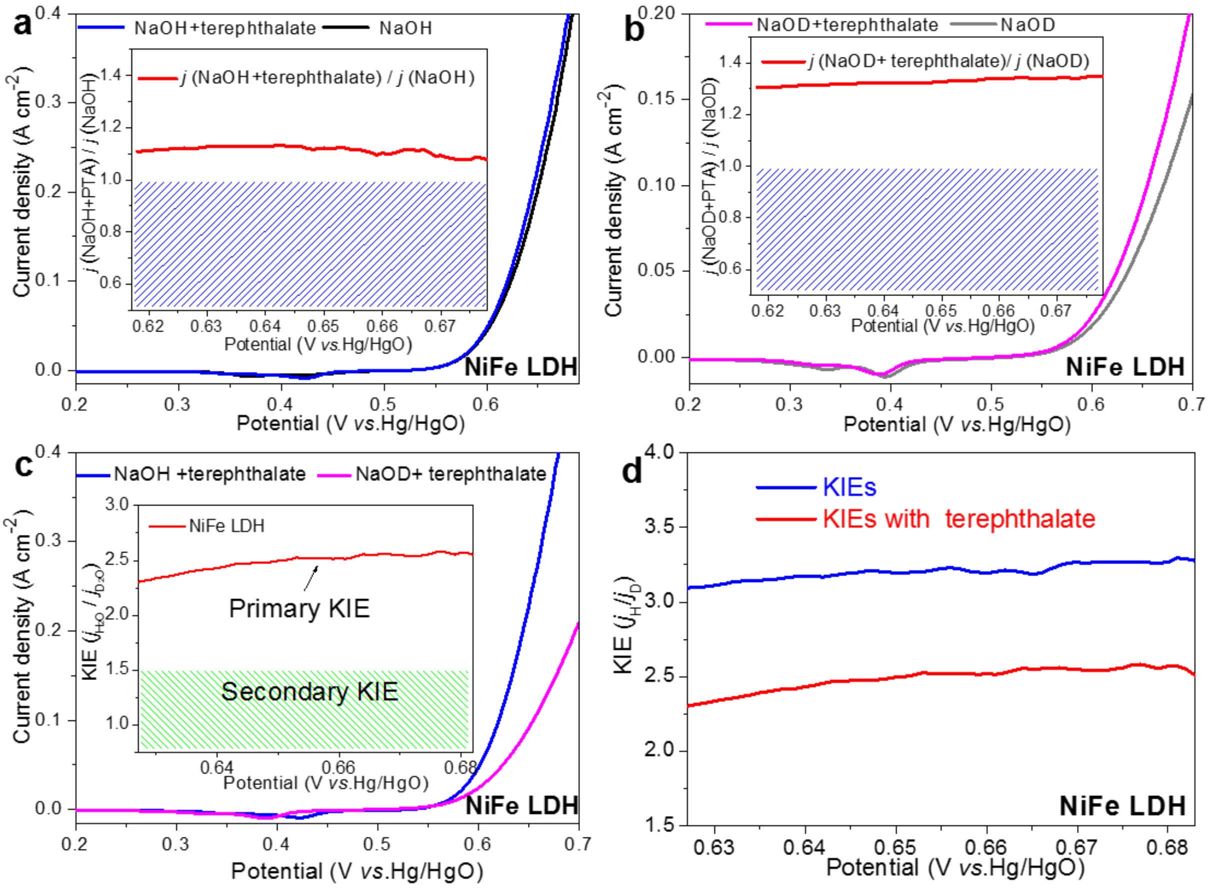

Supplementary Figure 15. Kinetic Isotope Effects characterizations of **NiFe LDH/NF**.

a LSV curves of **NiFe LDH/NF** in aqueous 1.0 M NaOH/H_2O solution and 1.0 M NaOH/H_2O solution with 0.3 M anhydrous disodium terephthalate. The inset exhibits the quotients of corresponding current densities vs potentials.

b LSV curves of **NiFe LDH/NF** in aqueous 1.0 M NaOD/D_2O solution and 1.0 M NaOD/D_2O solution with 0.3 M anhydrous disodium terephthalate. The inset exhibits the quotients of corresponding current densities vs potentials.

c LSV curves of **NiFe LDH/NF** in aqueous 1.0 M NaOH/H_2O solution with 0.3 M anhydrous disodium terephthalate and 1.0 M NaOD/D_2O with 0.3 M solution with anhydrous disodium terephthalate. The inset exhibits the KIEs values vs potentials.

d The KIEs vs potentials of **NiFe LDH/NF** with and without terephthalate.

Supplementary Table

Supplementary Table 1. Comparison of OER activity of **NiFeCP/NF** prepared in this work with recently-reported catalysts in alkaline solution.

**Catalyst**	**Electrolyte**	**Current density (mA cm^{-2})**	**Overpotential (mV)**	**Tafel slope (mV dec^{-1})**	**Reference**
NiFeCP/NF	1 M KOH	10	188	29	This work
NiFeCP/NF	1 M KOH	50	214	29	This work
NiCo-UMOFNs/CF	1 M KOH	10	189	42	1
NiFe-UMNs/GC	1 M KOH	10	260	30	2
NiFe (MIL-53)/NF	1 M KOH	50	233	31.3	3
NiFe-MOF/NF	1 M KOH	10	240	34	4
NiFe-MOF/NF	1 M KOH	10	260	35	5
NiFe LDH/r-GO/NF	1 M KOH	10	195	39	6
$Fe_{0.5}Ni_{0.5}$@N-GR	1 M KOH	10	210	62.0	7
Ni_2P/GC	1 M KOH	10	290	47	8
$Ni_{60}Fe_{30}Mn_{10}$	0.5 M KOH	10	200	62	9
$Co_{0.37}Ni_{0.26}Fe_{0.37}O$	1 M KOH	10	232	37.6	10
$(Ni_{0.5}Fe_{0.5})_2P$/NF	1 M KOH	50	251	57	11
$Ni_{0.83}Fe_{0.17}(OH)_2$/GC	1 M KOH	10	245	61	12
NiFeMo/NF	1 M KOH	10	238	35	13
$Ni_xFe_{1-x}Se_2$-DO/NF	1 M KOH	10	195	28	14
NiFe LDH-NS@DG	1 M KOH	10	210	52	15

Supplementary Notes

Supplementary Note 1. pH-dependence characterizations

The LSV or CV were recorded with a scan rate of 5 mV s^{-1} and iR compensated (80%) in potassium hydroxide solutions with different value of pH. The reaction order of the concentration for OH^- can be calculated according to **eqn. 1**, where *j* is the current density (mA) at a certain overpotential of η, $[OH^-]$ is the concentration of hydroxide in electrolytes (mol L^{-1}).

$$\rho_{[OH^-]} = \left[\frac{\partial \log j}{\partial \log [OH^-]} \right]_\eta \qquad 1$$

According to the dissociation equilibrium of water, $14 = (-\log[OH^-]) + (-\log[H^+])$, then the reaction order of $[OH^-]$ can be calculated according to **eqn. 2**

$$\rho_{[OH^-]} = \left[\frac{\partial \log j}{\partial (-14 + \mathrm{pH})} \right]_\eta = \left[\frac{\partial \log j}{\partial \mathrm{pH}} \right]_\eta \qquad 2$$

Supplementary Note 2. Kinetic Isotope Effects characterizations

Kinetic isotope effects (KIEs) were studied via electrochemical methods. The LSV or CV were recorded with a scan rate of 5 mV s^{-1} and iR compensated (80%), the experiments were carried out in 1.0 M NaOH aqueous solution and 1.0 M NaOD D_2O solution, the corresponding current densities at a certain overpotential of η were abbreviated as j_{H2O} and j_{D2O}. Then, the $KIEs_{H/D}$ was defined as **eqn. 3**.

$$KIEs_{H/D} = \left[\frac{k_{H_2O}}{k_{D_2O}} \right]_\eta = \left[\frac{j_{H_2O}}{j_{D_2O}} \right]_\eta \qquad 3$$

According to Arrhenius equation, the rate constant of a chemical reaction is depended on the activation energy for the reaction. For an electrochemical reaction, according to Butler–Volmer equation, the activation overpotential is the potential difference above the equilibrium value required to produce a current, which depends on the activation energy of the redox event.[16] Because of that the catalytic activity will raise with the overpotential adding on the catalyst increasing, both **NiFeCP** and **NiFe LDH** exhibit negligible pH-dependent OER kinetics, thence, for KIEs measurements,

the current densities j_{H2O} and j_{D2O} should be contrasted at the same overpotential.

The overpotential can be corrected by **eqn. 4** for the measurement in 1.0 M NaOH aqueous solution.

$$\eta^{H_2O} = E_{RHE} - 1.229\,\mathrm{V}_{RHE} = E^{read}_{Hg/HgO} + 0.059\,pH + E^{H}_{Hg/HgO} - 1.229\,\mathrm{V}_{RHE} \quad 4$$

Where $E^{read}_{Hg/HgO}$ is the potential read by using as the Hg/HgO reference electrode; $E^{H}_{Hg/HgO}$ is the equilibrium potential for Hg/HgO couple in the NHE scales (normal hydrogen electrode, 0.0977 V).[16,17]

As the equilibrium potentials for the D_2/D^+ reaction, and the O_2/D_2O electrochemical reactions are different from their equivalent reactions in H_2O, overpotentials should be corrected to the RDE (reversible "deuterium" electrode in D_2O) scales before being used to calculate the KIEs.[18] The different free energy of formation ($\Delta_{formation}$ G) of H_2O (−237.18 kJ/mol) vs D_2O (−243.49 kJ/mol) leads to different equilibrium potentials of 1.229 V_{RHE} and 1.262 V_{RDE} for water oxidation.[18,19]

The overpotential can be corrected by **eqn. 5** for the measurement in 1.0 M NaOD/D_2O solution.

$$\eta^{D_2O} = E_{RDE} - 1.262\,\mathrm{V}_{RDE} = E^{read}_{Hg/HgO} + 0.059\,pD + E^{D}_{Hg/HgO} - 1.262\ \mathrm{V}_{RDE} \quad 5$$

Where $E^{read}_{Hg/HgO}$ is the potential read by using as a Hg/HgO reference electrode (vs. Hg/HgO). $E^{D}_{Hg/HgO}$ is the equilibrium potential for Hg/HgO couple in the NDE scales (normal "deuterium" electrode). The equilibrium potential for the deuterium couple (D_2/D^+) is different from that of the proton couple (H_2/H^+) (−0.013 V),[19] which means the difference of NDE and NHE scales is −0.013 V, then the equilibrium potential for Hg/HgO couple for NDE scales is (0.0977 - 0.013) V.

Because of that 1.0 M NaOH/ H_2O and 1.0 M NaOD/D_2O were used as electrolytes, we used the concentration of OH^- and OD^- to correct the overpotential. As known, pK_{D2O} = pOD + pD = 14.87; pK_{H2O} = pOH + pH = 14,[20] then the **eqn. 4** and **5** become:

$$\eta^{H_2O} = E_{RHE} - 1.229\,\mathrm{V}_{RHE} = E^{read}_{Hg/HgO} + 0.059(14 - pOH) + E^{H}_{Hg/HgO} - 1.229\,\mathrm{V}_{RHE} \quad 6$$

$$\eta^{D_2O} = E_{RDE} - 1.262\,\mathrm{V}_{RDE} = E^{read}_{Hg/HgO} + 0.059(14.84 - pOD) + E^{D}_{Hg/HgO} - 1.262\,\mathrm{V}_{RDE} \quad 7$$

Considering that the concentration of OH^- and OD^- in 1.0 M NaOH/ H_2O and 1.0 M NaOD/D_2O electrolytes are same (pOD ≈ pD), then at equal potential read by using as a Hg/HgO reference electrode ($E^{read}_{Hg/HgO}$), the difference of overpotentials in 1.0 M NaOD/D_2O and 1.0 M NaOH/H_2O ($\eta^{D_2O} - \eta^{H_2O}$) is approximately 0.003 V, which can be neglected, therefore, we compared the current densities j_{H2O} and j_{D2O} at the same potential by using as a Hg/HgO reference electrode (vs Hg/HgO) for the calculation of KIEs.

Supplementary Note 3. Proton inventory studies

Proton inventory were studied via electrochemical methods. The LSV or CV were recorded with a scan rate of 5 mV s^{-1} and iR compensated (80%), the experiments were carried out in mixed solutions of 1.0 M NaOH in H_2O and 1.0 M NaOD in D_2O with different ratios, the corresponding current densities at given fractional deuteration concentrations n (n = [D_2O]/([D_2O]+[H_2O])) and at a certain overpotential of η were abbreviated as j_n. j_0 is the corresponding measured current density in solutions without deuterium (D_2O and OD^-; j_0 = j_{H2O}). For the overpotential, we did the same treatment as KIEs measurements, by directly comparing the j_n and j_0 at the same potential vs. Hg/HgO.

$$\frac{j_n}{j_0} = \left(1 - n + n\phi\right) Z^n \quad 8$$

Eqn. 8 was used to fit the proton inventory data, which was derived from the Kresge-Gross-Butler equation (**eqn. 9**):[21-23]

$$k_n = k_0 \left[\frac{\prod_{i=1}^{x} \left(1 - n + n\phi_{Ti}\right)}{\prod_{i=1}^{x} \left(1 - n + n\phi_{Ri}\right)} \right] Z^n \quad 9$$

where k_0 is the kinetic rate constant in solution without deuterium, k_n is the kinetic rate constant in a solution containing a mole fraction of D_2O of n, x is the number of hydrogenic sites in the reactant or transition state, ϕ_{Ti} and ϕ_{Ri} are the isotopic

fractionation factor for hydrogenic site in the transition- and reactant-state, respectively. Z^n reflects the Z-effect, when Z = 1, there are no Z-sites that contribute to the isotope effect; when Z > 1, then the Z-sites contribute an inverse isotope effect; and when Z < 1 then Z-sites contribute a normal isotope effect.[24-26]

In our studies, the pronounced kinetics isotope effect occurs at a single concerted proton-coupled electron transfer step for both **NiFeCP** and **NiFe LDH**, which are considered as a step only single hydrogenic site involved for pronounced kinetics isotope effect, the x was token as 1, therefore, **eqn. 9** can be reduced to:

$$k_n = k_0 \frac{1-n+n\phi_T}{1-n+n\phi_R} Z^n \qquad 10$$

To simplify the model, we assume that the reactant-state fractionation factor for the OH— attached to metal-oxo by nuclear attack is near unity, and therefore do not make important contributions to the solvent isotope effect ($\varnothing_R$ ~ 1),[22] then **eqn. 10** becomes:

$$k_n = k_0 \left(1-n+n\phi\right) Z^n \qquad 11$$

Where ∅ is the isotopic fractionation factor for hydrogenic site involved in the rate determine step in the transition-state. Combining **eqn. 3** and **11** produce the **eqn. 12** and **eqn. 8**:

$$j_n = j_0 \left(1-n+n\phi\right) Z^n \qquad 12$$

Eqn. 8 and **12** represent the isotope effect arises from a combination of pronounced isotope effect at a few sites (i.e., these sites have ∅ values that are quite different than unity), and from a Z-effect (i.e. these sites have ∅ values that are very close to unity individually but has an aggregate isotope effect as a whole).[24]

The currents at potentials of 0.667 V, 0.662 V, 0.657 V, 0.652 V, 0.647 V, 0.642 V, 0.637 V, 0.632 V, 0.627 V, 0.622 V, 0.617 V and 0.612 V were taken for the calculation of **NiFeCP**. The currents at potentials of 0.668 V, 0.663 V, 0.658 V, 0.653 V, 0.648 V, 0.643 V, 0.638 V, 0.633 V, 0.628 V, 0.623 V, 0.618 V were taken for the calculation of **NiFe LDH**.

Supplementary Note 4. Determination of the reaction order for phosphate

The reaction order of additional bases ($\rho_{[B]}$) can be calculated according to **eqn. 13**, where j is the current density (mA), [B] is the concentration of additional base (mol L^{-1}).

$$\rho_{[B]} = \left[\frac{\partial \log j}{\partial \log [B]} \right]_{\eta} \qquad 13$$

For the base-dependent pathway of water oxidation, the water oxidation reaction rate k_{cat} can be composed of two components k^{H2O} (rate constant for unassisted water oxidation) and k^{B} (base assist water oxidation, meaning phosphate act as the proton acceptor during the rate determining step in this work) (**eqn. 14**).[27,28]

$$k_{cat} = k^{H_2O} + k^{B}[B] \qquad 14$$

And k_{cat} stands for the catalytic turnover frequency (TOF, s^{-1}). k_{cat} is positive linear correlation with current density for catalyst modified electrodes, as describe in **eqn. 15**, where *j* is the current density (A cm^{-2} or C s^{-1} cm^{-2}), *A* is the surface area (cm^2) of the electrode; *F* is the Faraday constant (96485 C mol^{-1}); and *Γ* is the electroactive amount (mol cm^{-2}).[17,29]

$$k_{cat} = \left[\frac{jA}{4F\Gamma A} \right]_{\eta} \qquad 15$$

The current density will be linear relationship to the concentration of base for the Atom-Proton Transfer (APT) assisted electrode catalytic water oxidation, as describe in **eqn. 16**. According to **eqn. 13**, which means that it should a 1st order reaction of additional base,

$$\left[\frac{jA}{4F\Gamma A} \right]_{\eta} = k^{H_2O} + k^{B}[B] \qquad 16$$

$\rho_{[phosphate]}$ was measured according to **eqn. 13**. The K_3PO_4 solutions with different concentration of 0.75 M,1 M,1.25 M ,1.5 M and 1.75 M were prepared, and 1.75 M K_3PO_4 has a pH of 12.65 measured with a pH meter. The pH of K_3PO_4 with concentration of 0.75 M, 1 M, 1.25 M and 1.5 M were adjusted to 12.65 by adding potassium hydroxide.

Supplementary Methods

Materials

Nickel nitrate hexahydrate ($Ni(NO_3)_2·6H_2O$, 99%), Iron nitrate nonahydrate ($Fe(NO_3)_3·9H_2O$, 99%), Terephthalic acid (99%), Disodium Terephthalate (> 99%), Potassium phosphate tribasic (K_3PO_4, > 99%), KOH (98%) and N, N-dimethylformamide (DMF) were purchased from Aladdin Reagent, Shanghai, China. All aqueous solutions were prepared with high-purity deionized water (Milli-Q, resistance 18 MΩ cm^{-1}). Deuterated electrolytes were prepared using D_2O (Sigma-Aldrich, 99.9% atom % D), and 40 wt% NaOD solution in D_2O (Sigma-Aldrich, 99 atom % D). All chemicals were used directly without further purification. The thickness of nickel foam (**NF**) is 1.0 mm, bulk density is 0.5 g cm^{-3}.

Physical characterization

The morphology and composition of the fabricated films were characterized by field emission scan electron microscopy (FE-SEM, Carl Zeiss Supra 55, operated at 15 kV) equipped with energy dispersive X-ray (EDX) microanalysis (Oxford EDS Inca Energy Coater 300, operated at 20 kV). High-angle annular dark-field scanning transmission electron microscopy (HAADF-STEM) images and energy-dispersive X-ray spectroscopy (EDS) mapping were collected on a FEI Talos F200X field-emission transmission electron microscope operated at 200 kV. The samples for TEM spectrum were detached by sonication from the **NiFeCP/NF**. The detached powder was added into 5 mL ethanol and sonicated for 30 min. The suspensions were added drop-wise onto a carbon-coated copper grid and dried in air before testing. The surface composition of the electrode films was investigated using X-ray photoelectron spectroscopy (XPS) on an ESCALAB 250Xi (Thermo Scientific™). Raman spectroscopy was measured by DXR Microscope (Thermo Fisher™), Fourier transform infrared spectra (FT-IR) was measured by Nicolet 6700 Flex (Thermo Fisher™). X-ray diffraction (XRD) was measured by SmartLab (Rigaku™). The pH of electrolyte was measured 914 pH/conductometer (Metrohm™)

Supplementary References

1. Zhao, S., et al. Ultrathin metal–organic framework nanosheets for electrocatalytic oxygen evolution. Nat. Energy **1**, 16184 (2016).
2. Hai, G., et al. High-performance oxygen evolution catalyst using two-dimensional ultrathin metal-organic frameworks nanosheets. Nano Energy **44**, 345-352 (2018).
3. Sun, F., et al. NiFe-Based Metal–Organic Framework Nanosheets Directly Supported on Nickel Foam Acting as Robust Electrodes for Electrochemical Oxygen Evolution Reaction. Adv. Energy Mater. **8**, 1800584 (2018).
4. Duan, J., Chen, S. & Zhao, C. Ultrathin metal-organic framework array for efficient electrocatalytic water splitting. Nat. Commun. **8**, 15341 (2017).
5. Li, J., et al. Low-Crystalline Bimetallic Metal–Organic Framework Electrocatalysts with Rich Active Sites for Oxygen Evolution. ACS Energy Lett., 285-292 (2018).
6. Long, X., et al. A strongly coupled graphene and FeNi double hydroxide hybrid as an excellent electrocatalyst for the oxygen evolution reaction. Angew. Chem. **53**, 7584-7588 (2014).
7. Peitao Liu, et al. Self-Powered Water-Splitting Devices by Core–Shell NiFe@N-Graphite-Based Zn–Air Batteries. Adv. Funct. Mater. **28**, 1706928 (2018).
8. Lucas-Alexandre Stern, Ligang Feng, Song, F. & Hu, X. Mesoporous Ni60Fe30Mn10-alloy based metal/metal oxide composite thick films as highly active and robust oxygen evolution catalysts. Energy Environ. Sci. **9**, 540 (2016).
9. Eric Detsi, et al. Mesoporous Ni60Fe30Mn10-alloy based metal/metal oxide composite thick films as highly active and robust oxygen evolution catalysts. Energy Environ. Sci. **9**, 540 (2016).
10. Chen, W., et al. In Situ Electrochemical Oxidation Tuning of Transition Metal Disulfides to Oxides for Enhanced Water Oxidation. ACS Cent Sci **1**, 244-251 (2015).
11. Yu, J., Cheng, G. & Luo, W. Hierarchical NiFeP microflowers directly grown on Ni foam for efficient electrocatalytic oxygen evolution. J. Mater. Chem. A, **5**, 11229 (2017).
12. Zhou, Q., et al. Active-Site-Enriched Iron-Doped Nickel/Cobalt Hydroxide Nanosheets for Enhanced Oxygen Evolution Reaction. ACS Catal. , 5382-5390 (2018).
13. Qin, F., et al. Trimetallic NiFeMo for Overall Electrochemical Water Splitting with a Low Cell Voltage. ACS Energy Lett. **3**, 546-554 (2018).
14. Xu, X., Song, F. & Hu, X. A nickel iron diselenide-derived efficient oxygen-evolution catalyst. Nat Commun **7**, 12324 (2016).
15. Jia, Y., et al. A Heterostructure Coupling of Exfoliated Ni-Fe Hydroxide Nanosheet and Defective Graphene as a Bifunctional Electrocatalyst for Overall Water Splitting. Adv. Mater. **29**, 1700017 (2017).
16. Bard, A.J. & Faulkner, L.R. Electrochemical methods: fundamentals and applications, (Wiley New York, 1980).
17. Bamford, C.H., Tipper, C.F.H. & Compton, R.G. Electrode Kinetics: Principles and Methodology: Principles and Methodology, (Elsevier, 1986).
18. Malko, D. & Kucernak, A. Kinetic isotope effect in the oxygen reduction reaction (ORR) over Fe-N/C catalysts under acidic and alkaline conditions. Electrochem. Commun. **83**, 67-71 (2017).
19. Bard, A. Standard potentials in aqueous solution, (Routledge, 2017).
20. Bates, R.G., Robinson, R.A. & Covington, A.K. pK values for D2O and H2O. J. Chem. Educ. **44**, 635 (1967).

21. Fitzpatrick, P.F. Combining solvent isotope effects with substrate isotope effects in mechanistic studies of alcohol and amine oxidation by enzymes. Biochimica et Biophysica Acta (BBA) - Proteins and Proteomics **1854**, 1746-1755 (2015).
22. Kohen, A. & Limbach, H.-H. Isotope effects in chemistry and biology, (cRc Press, 2005).
23. Haschke, S., et al. Direct oxygen isotope effect identifies the rate-determining step of electrocatalytic OER at an oxidic surface. Nat. Commun. **9**, 4565 (2018).
24. Liu, Y. & McCrory, C.C.L. Modulating the mechanism of electrocatalytic CO2 reduction by cobalt phthalocyanine through polymer coordination and encapsulation. Nat. Commun. **10**, 1683 (2019).
25. Schowen, R.L. The use of solvent isotope effects in the pursuit of enzyme mechanisms. J. Labelled Compd. Radiopharm. **50**, 1052-1062 (2007).
26. Venkatasubban, K.S. & Schowen, R.L. The Proton Inventory Techniqu. Critical Reviews in Biochemistry **17**, 1-44 (1984).
27. Chen, Z., et al. Concerted O atom–proton transfer in the O—O bond forming step in water oxidation. Proc. Natl. Acad. Sci. U.S.A. **107**, 7225 (2010).
28. Wang, L., et al. A nickel (II) PY5 complex as an electrocatalyst for water oxidation. J. Catal. **335**, 72-78 (2016).
29. Wang, J., et al. In situ formation of molecular Ni-Fe active sites on heteroatom-doped graphene as a heterogeneous electrocatalyst toward oxygen evolution. Sci. Adv. **4**(2018).
